# Evaluation of Antibacterial Effects of Fissure Sealants Containing Chitosan Nanoparticles

**DOI:** 10.1155/2021/8975948

**Published:** 2021-08-16

**Authors:** Sedighe Sadat Hashemi kamangar, Houtan Zareian, Abbas Bahador, Maryam Pourhajibagher, Zahra Bashareh, Sara Valizadeh

**Affiliations:** ^1^Department of Operative Dentistry, International Campus, Dental School, Tehran University of Medical Sciences, Tehran, Iran; ^2^International Campus, Dental School, Tehran University of Medical Sciences, Tehran, Iran; ^3^Department of Medical Microbiology, Faculty of Medicine, Tehran University of Medical Sciences, Tehran, Iran; ^4^Dental Research Center, Dentistry Research Institute, Tehran University of Medical Sciences, Tehran, Iran; ^5^Restorative Dentistry Department, School of Dentistry, Tehran University of Medical Sciences, Tehran, Iran

## Abstract

**Objectives:**

The present study evaluated the antimicrobial effects of fissure sealants containing chitosan nanoparticles.

**Materials and Methods:**

Antibacterial effect of Master Dent fissure sealant alone and after incorporating chitosan nanoparticles was evaluated on *Streptococcus mutans*, *sanguis*, and *Lactobacillus acidophilus*. Biofilm growth was evaluated by determining colony counts. Antimicrobial effect was determined on days 3, 15, and 30 by counting microbial colonies using eluted components test. One-way ANOVA, Tukey HSD tests, *t* test, and two-way ANOVA were used for statistical analyses (*α* = 0.05).

**Results:**

Biofilm inhibition test showed that fissure sealant containing 1 wt.% chitosan decreased colony counts significantly (*P* < 0.05). Eluted components test with *S. mutans* and *sanguis* showed significant decrease in colony counts during the first 15 days in chitosan containing group; however, from day 30, antimicrobial activity decreased noticeably, with no significant difference from control group (*P* > 0.05). Antimicrobial activity against *L. acidophilus* was maintained in chitosan group up to 30 days, and decrease in colony counts was significant (*P* < 0.05).

**Conclusion:**

According to the results of this study, incorporation of 1 wt.% chitosan into fissure sealant induced an antimicrobial activity. Antibacterial effect on *L. acidophilus* persisted for longer time (30 days) compared to the two other bacterial species (15 days).

## 1. Introduction

Despite advances in identifying cariogenic factors and caries prevention techniques, dental caries is still an oral condition due to biofilms with a pandemic distribution pattern. The pits and fissures on the occlusal surfaces of permanent teeth with deep and narrow topography are considered a challenge for the mechanical cleaning of these areas [1]. Therefore, these surfaces are very susceptible to the invasion of oral microflora and their metabolic byproducts. It has been reported that sealing pits and fissures can be a preventive measure against bacterial invasion by creating a physical protective barrier [2].

Previous studies have shown that resin-based sealers are better than other materials in terms of retention and sealing ability and can decrease the risk of new caries up to 18% in high-risk individuals. They are superior to glass ionomer in terms of retention, with similar results in terms of prevention [3]. Although sealers are widely used to prevent dental caries, they exhibit a high failure rate. Bacterial colonization beneath the sealants and demineralization of teeth are among the important factors which are attributed to this issue [4, 5].

In vitro studies have shown that despite fissure sealants' good sealing ability, microleakage is observed around them, especially after thermocycling, associated with the adhesion of cariogenic bacteria around the sealant and the subsequent failure to prevent caries [6]. Some clinical studies indicate that sealants with the compromised retention could reduce the longevity of fissure sealant. In the other words, the clinical success of sealants in preventing caries is strongly associated with their retention [7].

The oral cavity is associated with significant physical and chemical challenges for resin-based sealants due to its high moisture level, thermal changes, and pH. In addition, acids produced by acidogenic bacteria, such as *S. mutans*, are inherently present in the saliva and can endanger the materials' properties over time, leading to the demineralization of the tooth structure adjacent to a sealant [8]. Abrasion and physical detachment of sealers over time increases the risk of recurrent caries. Therefore, incorporating antibacterial agents into sealants to produce materials with therapeutic effects and improved biologic properties, especially in the long term, has been considered [9, 10].

Different antibacterial agents that have been incorporated into dimethacrylate resin-based sealants have not been associated with much success. Fluoride and chlorhexidine are the most common materials added to sealants, which have exhibited strong antibacterial activity initially but have lost such activity rapidly over time. Besides, they have endangered the sealants' mechanical properties by affecting their mechanical properties [11].

A natural product recently added to restorative materials as an antibacterial agent is chitosan, a biopolymer derived from the shrimp outer crust and other crustaceans. Since chitosan has a positive charge, it binds to the bacterial cell wall or cell membrane to exert its bactericidal effect [12]. Chitosan exerts its most significant effect on gram-positive bacteria, such as *S. sanguis*, *S. mitis*, and *S. salivarius*. The advantages of chitosan include low cost, prevention of demineralization, prevention of biofilm formation, stimulation of salivary flow, and a lack of immune system stimulation [13].

Dental caries is due to the acids produced by bacteria, especially *S. mutans* and *Lactobacillus species*. *S. mutans*, which is found in large amounts in the cariogenic plaque, has anaerobic activity at pH = 5.5 and produces organic acids. *L. acidophilus* is another bacterial species that causes demineralization. Another microorganism found in noncariogenic plaque is *S. sanguis*, whose presence in the plaque indicates a low cariogenic activity of the plaque [14].

Only a small number of studies are available on incorporating chitosan nanoparticles into fissure sealants, and there is still no consensus on the effective concentration of this material in the sealant for maximum antimicrobial activity, with no adverse effect on its physical and mechanical properties. Besides, most previous studies have only evaluated *S. mutans*, and the long-term antibacterial effect has not been evaluated. Therefore, the present study aimed to determine the antibacterial effect of incorporating chitosan nanoparticles into a sealant and preservation of the antimicrobial activity over time with 1 wt.% concentration on *S. mutans*, *S. sanguis*, and *L. acidophilus* using the biofilm inhibition test and eluted components test.

## 2. Materials and Methods

### 2.1. Preparation of Nanocomposite Containing Chitosan

A pilot study was carried out to determine the weight percentage of nanoparticles, the mixing method, ensuring the curing process, fissure sealant viscosity, and particles' distribution. Commercial nanochitosan powder (Sigma-Aldrich, USA) was used to produce this composite. Master Dent (Dentonics, Inc., Monroe, NC, USA) fissure sealant paste was mixed with chitosan nanoparticles at 1 wt.% in a Sonoplus mixer (Ultrasonic, Bandelin, Germany) at high speed (3500 rpm) in a dark environment for 1 minute. Finally, EDAX-SEM was used to observe nanoparticles' distribution in the composite to ensure homogeneous distribution of nanoparticles in the composite.

### 2.2. Preparation of Bacterial Suspensions

Standard strains of *S. mutans* (ATCC 35668), *S. sanguis* (ATCC 10556), and *L. acidophilus* (ATCC 314) were procured from the Iranian National Genetics Reserve Center and cultured on Mutans-Mitis-Salivarius valinomycin (Merck, Darmstadt, Germany) specific culture medium under aerobic conditions in the presence of 5% CO_2_, MM10 sucrose agar (Merck, Darmstadt, Germany) under aerobic conditions in the presence of 5% CO_2_, and MRS (Merck, Darmstadt, Germany) under anaerobic conditions, respectively, and incubated at 37°C for 48 hours.

The bacteria were cultured in the brain-heart infusion (BHI) broth (Merck, Darmstadt, Germany) to prepare microbial suspensions and kept at 37°C under each bacterial species' growth conditions. Then, a concentration of 1.5 × 10^8^ CFU/mL (optical density [OD] at 600 nm: 0.88–0.1) was prepared from each bacterial species in the logarithmic growth phase using a spectrophotometer, and the concentration was confirmed by culture.

### 2.3. Biofilm Inhibition Test and Evaluation of Colony Counts

To form microbial biofilms, 300 *µ*L of the bacterial suspension at 1.5 × 10^8^ CFU/mL was added to the composite disks in the microplate wells and incubated at 37°C for 48 hours in terms of each bacterial species' growth conditions. The composite disks were then rinsed with 5 mL of a sterile normal saline solution under aseptic conditions and placed in sterilized microtubes containing 1 mL of BHI broth (Merck, Darmstadt, Germany) to eliminate planktonic bacteria with loose attachment. The microtubes containing the disks were vortexed at high speed for 1 minute to separate bacterial biofilms from the composite disk surfaces.

After preparing serial dilutions of the bacterial biofilm in 96-well microplates, 10 *µ*L of each dilution was inoculated into the BHI agar (Merck, Darmstadt, Germany) and cultured using the spreading method. The plates were incubated at 37°C for 24 hours in terms of each bacterial species' growth conditions, and each sample's CFU/mL was calculated using Miles et al.'s method [15].

### 2.4. Eluted Components Test

The composite disks were placed in microtubes containing 1 mL of artificial saliva. On days 3, 15, and 30, 450 *µ*L of the above artificial saliva was added to a new microtube containing 50 *µ*L of bacterial suspension with a final concentration of 1.5 × 10^6^ CFU/mL. The microtubes were shaken at 300 rpm at 37°C for 24 hours in terms of bacterial species. Finally, each sample's CFU/mL was determined through serial dilutions in 96-well microplates, followed by spread culturing in the BHI agar environment using Miles et al.'s method [15].

### 2.5. Statistical Analysis

Several statistical tests were used for data analysis. First, *t* test was used to analyze the biofilm inhibition test. Two-way ANOVA was used to analyze the eluted components test. One-way ANOVA, post hoc Tukey tests, and *t* test were used for two-by-two comparisons of the groups. Statistical significance was set at *P* < 0.05.

## 3. Results

### 3.1. Biofilm Inhibition Test and Evaluation of Colony Counts

Table 1 presents the means and standard deviations of the colonies formed at 1 wt.% of chitosan compared to the control group in the biofilm inhibition test for the three bacterial species evaluated. In all the groups, the maximum colony counts in the biofilm inhibition test of the three bacterial species were related to the 1 wt.% concentration of chitosan, with the lowest colony counts in the control group. Besides, the three bacterial species' colony growth in the 1 wt.% chitosan group was significantly less than the control groups. Table 1 presents the *P* values of the three bacterial species.

### 3.2. Eluted Components Test

Table 2 presents the means and standard deviations of the colony counts on days 3, 15, and 30 in the fissure sealant group containing chitosan and the control group for the three microorganisms. The results of this test in different bacterial species were as follows.

### 3.3. Lactobacillus acidophilus

The maximum colony counts were recorded with the 1 wt.% chitosan on the 15th day, and the minimum colony count was recorded in the control group on the 15th day. At the 1 wt.% concentration of chitosan, the colony counts decreased on the 3rd and 15th days but increased on the 30th day. The colony counts increased over time in the control group.

Two-way ANOVA showed that the factors of day (*P*=0.025*|*), chitosan concentration (*P*=0.0001), and the interaction of days and concentrations (*P*=0.053) significantly affected the colony counts.

Since the interaction between the days and concentration was not significant, and there were only two chitosan concentrations in the present study, which exhibited significant differences, post hoc Tukey tests were used for two-by-two comparisons of days only. The results showed no significant differences between day 3 and the two other days (*P* > 0.05); a significant difference was noted between the 15th and 30th days (*P*=0.032).

**Figure 1:**
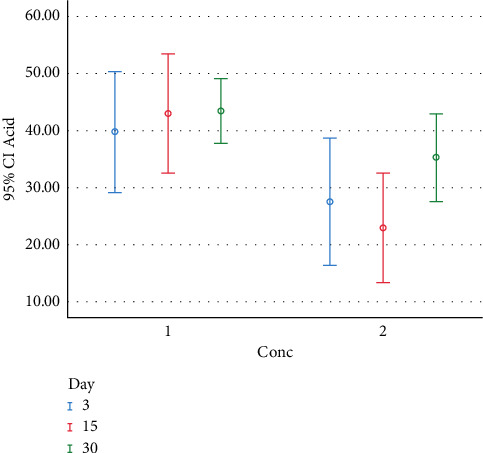
The distribution and colony counts formed at different study intervals (days) and concentrations (Conc) for *L. acidophilus* in the eluted components test (Conc. 1 : 0% chitosan; Conc. 2 : 1% chitosan).

Figure 1 presents the distribution and number of colonies found on different days with different concentrations of *L.acidophilus* using the eluted components test.

### 3.4. *S. mutans* and *S. sanguis*

The highest decrease in the colony counts of *S. mutans* and *S. sanguis* occurred with the 1 wt.% concentration of chitosan on day 15. The lowest colony count was related to the control group (0% chitosan) on day 30. At 1 wt.% concentration of chitosan on days 3 and 15, the colony counts decreased, increasing on day 30. The colony counts increased over time in the control group.

Two-way ANOVA showed that the factors of day (*P*=0.006 for *S. mutans* and *P*=0.001 for *S. sanguis*) and concentration (*P*=0.0001) and the interaction between day and concentration (*P*=0.039 for *S. mutans* and *P*=0.005 for *S. sanguis*) significantly affected the colony counts of these two bacterial species.

Since the interaction between the day and concentration factors was significant, one-way ANOVA was used to determine the differences between each concentration on different days. The results showed no significant differences in colony counts between different days in the control group (*P* > 0.05); however, at 1 wt.% concentration, there were significant differences between different days in both bacterial species (*P*=0.007 for *S. mutans* and *P*=0.002 for *S. sanguis*). Therefore, post hoc Tukey tests were used in the 1% chitosan group for two-by-two comparisons of the colony counts on the study days. The results showed a significant increase in colony counts on day 30 than other days; however, the colony counts were not significantly different between days 3 and 15 (*P* > 0.05).

**Figure 2:**
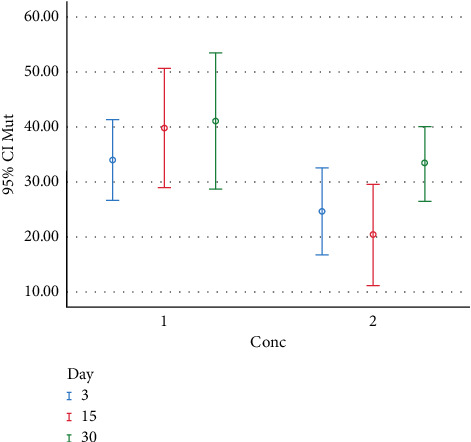
The distribution and colony counts formed at different study intervals (days) and concentrations (Conc) for *S. mutans* in the eluted components test (Conc. 1 : 0% chitosan; Conc. 2 : 1% chitosan).

**Figure 3:**
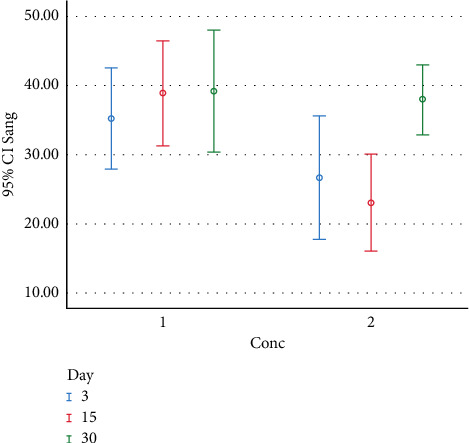
The distribution and colony counts formed at different study intervals (days) and concentrations (Conc.) for *S. sanguis* in the eluted components test (Conc. 1 : 0% chitosan; Conc. 2 : 1% chitosan).

*t* test was used to compare colony counts at different concentrations of chitosan on the study days. The results showed a significant difference between the colony group and 1% chitosan group between day 3 and day 15 (*P* < 0.05). However, on day 30, there was no significant difference between the control and 1% chitosan groups (*P* > 0.05). Figures 2 and 3 present the distribution and number of colonies on different days with different concentrations (Conc) of chitosan for *S. mutans* and *S. sanguis* in the eluted components test.

## 4. Discussion

Resin sealants effectively prevent caries progression; however, the number of sound sealants decreases over time (73% after five years). Therefore, their protective role is adversely affected. As a result, recently, great emphasis has been placed on the use of antibacterial agents with nanotechnology in sealants' composition to provide antibacterial properties and prevent biofilm formation around sealants [16].

In the present study, the antibacterial effect of incorporating chitosan nanoparticles into fissure sealants and preserving its antimicrobial activity over time was evaluated at its 1% concentration on *S. mutans*, *S. sanguis*, and *L. acidophilus*. The overall results showed that incorporating chitosan nanoparticles at 1% concentration into fissure sealant composites conferred antimicrobial properties to the fissure sealant.

In the biofilm inhibition test, the colony counting results in the control and chitosan-containing nanocomposite showed that chitosan nanoparticles significantly decreased *S. mutans*, *L. acidophilus*, and *S. sanguis* colonies; i.e., the highest antimicrobial activity was observed in the nanochitosan composite groups against the three bacterial species. The biofilm inhibition film test is essential in that, at a similar concentration of antimicrobial agents, the bacteria in the form of a biofilm are four times more resistant than the planktonic form [17].

In a study by Mahapoka et al., incorporation of chitosan whiskers to resin sealants at 1% 1.5%, 2%, and 2.5% concentrations improved the antibacterial properties of sealants compared to the control group; however, only sealants containing 2 and 2.5 wt.% of chitosan whiskers exhibited significantly higher antibacterial activity than the control group [18]. In the present study, 1 wt.% concentration of chitosan significantly increased the sealant's antibacterial activity. One of the reasons for such a discrepancy in the results might be the form of the chitosan added to the sealant, which was different in these two studies. Another reason might be the filler types used in sealants because Master Dent is a methacrylate resin-based sealant containing amorphous silica and a photoinitiator. Since silica (SiO_2_) has antimicrobial activity [19], chitosan with 1% concentration was effective, but the antimicrobial activity in the resin fissure sealant manufactured of 70 wt.% BisGMA, 41.9 wt.% TEGDMA, 0.86 wt.% 2-dimethylaminoethyl methacrylate, and 0.29 wt.% CQ was due to chitosan whiskers; therefore, at higher concentrations, there was no significant difference from the control group.

In a study by Choi et al. incorporating chitosan at 1, 1.5, and 5 wt.% into the test sealant did not result in any significant antibacterial activity, which is different from the results of many previous studies [17]. Such discrepancies between the results might be attributed to the chitosan type added to the sealant and the mechanism of manufacturing the chitosan nanofillers.

In a study by Sodagar et al. [19] on the antibacterial effect of a combination of Transbond XT and chitosan and ZnO with equal proportions at 1%, 5%, and 10% concentrations on *S. mutans*, *L. acidophilus*, and *S. sanguis*, the above combination was only effective at 5% and 10% concentration on *Streptococci* and on *L. acidophilus* at 10% concentration, which is different from the present study, in which the incorporation of 1% chitosan into Master Dent fissure sealant exerted an antibacterial effect on *S. sanguis* and *S. mutans*. The discrepancy in the results of these two studies might be due to differences in the type and viscosity of the composites used and combining chitosan and ZnO, preventing chitosan activity at low concentrations.

In the eluted components test, the antimicrobial effect of solutions containing nanoparticles possibly released from the nanocomposite disks was evaluated over time, which showed the persistence or decrease of antimicrobial activity over time. The present study showed significant decreases in the colony counts of *L. acidophilus* in the chitosan-containing fissure sealant group compared to the control group. The decrease in colony counts on day 15 was not significant compared to day 3. On day 30, the decrease in colony counts was significant compared to day 15; however, the difference from day 3 was not significant; i.e., the colony count increased from day 15 to day 30, equaling the colony counts on day 3. Therefore, it can be concluded that, in the chitosan group, the antimicrobial activity against *L. acidophilus* was preserved over time.

Furthermore, the present study showed that, in the chitosan group, the colony counts of *S. mutans* and *S. sanguis* decreased significantly on day 3 compared to the control group. The decrease in colony counts in the chitosan group was not significant on day 15 compared to day 3. However, on day 30, the significant increase in colony counts was significant, equaling the colony counts in the control group on day 30; i.e., in the chitosan group, the antimicrobial activity was preserved during the first 15 days, decreasing significantly from day 15 to day 30, with no significant difference from the control group. The antibacterial activity persisted only up to day 15. It appears that given the completion of polymerization over time and the decrease in flowability over time, nanoparticles' release and the antimicrobial activity decrease. However, further evaluations are necessary.

Mirhashemi et al. [20] showed that 5% and 10% concentrations preserved their antibacterial activity on *S. mutans* and *S. sanguis* up to 30 days. The 10% concentration exhibited a significant increase in antibacterial activity on *L. acidophilus* on day 30, contrasting the present study. Such a discrepancy, as discussed previously, might be due to differences in the type and viscosity of the composite used and the combination of chitosan and ZnO, preventing the activity of chitosan at low concentrations. Besides, it appears that considering the flowability of fissure sealants compared to conventional composites, the nanoparticles with antimicrobial activity accumulate on the surface during the curing process. Therefore, they provide significant antimicrobial activity even at low concentrations, such as 1%.

Rajabaia et al. [11] evaluated the effect of incorporating chitosan at 0%, 1%, 2%, 3%, 4%, and 5% concentrations to a fissure sealant (Clinpro; 3M ESPE, St. Paul, MN, USA) on *S. mutans* using direct contact test at 3-, 6-, 9-, 24-, and 48-hour and 1- and 3-month intervals. In contrast to the present study, in which 1% chitosan preserved its antibacterial activity up to 15 days, the minimum concentration of the antibacterial agent was 2%, and chitosan preserved its antibacterial activity in 2%, 3%, 4%, and 5% groups up to one month; however, after one month, the colony counts increased. The difference in the minimum concentration of antibacterial agents might be due to the type of filler used in the composite and the more remarkable persistence of antimicrobial activity due to the higher concentration of chitosan. Considering the antimicrobial effect observed after the incorporation of chitosan nanoparticles into the fissure sealant in the present study, preservation of the effect over time (at least 15 days), and the usefulness of the antimicrobial activity immediately after placing the fissure sealant in possibly destroying the residual bacteria in the fissure depths, it appears that the findings of the present study might be used to produce fissure sealants containing chitosan that are clinically useful, despite the limitations of this in vitro present study. It is necessary to evaluate the other properties like different mechanical and physical properties, adequate curing, sealing ability, and microleakage of this novel antibacterial fissure sealant in order to be sure that it would be appropriate for clinical use. Moreover, clinical condition in the oral environment is not easy to imitate in laboratory experiments.

It is suggested that studies be carried out on different concentrations of this material and the combination of fissure sealants with other nanoparticles to evaluate antimicrobial activity over more extended periods and the sealants' mechanical properties and bond strength.

## 5. Conclusions

The present study showed that chitosan nanoparticle-containing fissure sealant decreased the colony counts of all the three bacterial species tested in the biofilm inhibition test compared to the control group. The eluted components test showed that the fissure sealant containing chitosan nanoparticles significantly decreased *Lactobacillus* colonies up to 30 days and *Streptococcus* colonies up to 15 days compared to the control group. Therefore, under the limitations of the present study, it appears that incorporation of 1 wt.% chitosan into the fissure sealant was effective in inducing antibacterial activity.

## Figures and Tables

**Table 1:** The means and standard deviations of the colony counts (CFU/mm^2^) next to the fissure sealant containing 1% chitosan compared to the control group in the biofilm inhibition test for all the three bacterial species under study.

Chitosan concentration (%)	*mutans*		*sanguis*		*Lactobacillus*	
0	31.23 ± 3.23	*P*=0.001	30.97 ± 2.71	*P*=0.002	37.90 ± 3.08	*P*=0.021
1	11.63 ± 2.77		14.97 ± 2.35		27.93 ± 3.55	

**Table 2:** The means and standard deviations of the colonies formed in agar medium (CFU/mm^2^) on days 3 and 15 next to the fissure sealant containing 1% chitosan and the control group in the three bacterial species under study.

Chitosan concentration (%)	Time (days)	*mutans*	*sanguis*	*Lactobacillus*
0	3	33.93 ± 2.94 a	35.20 ± 2.96 a	39.73 ± 4.28 a
	15	39.73 ± 4.31 b	38.90 ± 3.03 b	42.96 ± 4.19 b
	30	40.96 ± 4.93 c	39.16 ± 3.57 c	43.40 ± 2.36 c

1	3	24.66 ± 3.17 d	26.66 ± 3.59 d	27.50 ± 4.49 d
	15	20.46 ± 3.69 e	23.00 ± 2.85 e	22.93 ± 3.88 e
	30	33.36 ± 2.74 d	37.93 ± 2.04 d	35.26 ± 3.10 d

^*∗*^The same superscript letters show nonsignificant differences.

## Data Availability

The data used to support the findings of this study are available from the corresponding author upon request.
